# Reviewing Data Integrated for PBPK Model Development to Predict Metabolic Drug-Drug Interactions: Shifting Perspectives and Emerging Trends

**DOI:** 10.3389/fphar.2021.708299

**Published:** 2021-10-28

**Authors:** Kenza Abouir, Caroline F Samer, Yvonne Gloor, Jules A Desmeules, Youssef Daali

**Affiliations:** ^1^ Division of Clinical Pharmacology and Toxicology, Department of Anesthesiology, Pharmacology, Intensive Care and Emergency Medicine, Geneva University Hospitals, Geneva, Switzerland; ^2^ Institute of Pharmaceutical Sciences of Western Switzerland (ISPSO), University of Geneva, Geneva, Switzerland; ^3^ Faculty of Medicine, University of Geneva, Geneva, Switzerland

**Keywords:** physiologically-based pharmacokinetics, drug-drug interaction, clinical setting, metabolism, precision dosing, transporters

## Abstract

Physiologically-based pharmacokinetics (PBPK) modeling is a robust tool that supports drug development and the pharmaceutical industry and regulatory authorities. Implementation of predictive systems in the clinics is more than ever a reality, resulting in a surge of interest for PBPK models by clinicians. We aimed to establish a repository of available PBPK models developed to date to predict drug-drug interactions (DDIs) in the different therapeutic areas by integrating intrinsic and extrinsic factors such as genetic polymorphisms of the cytochromes or environmental clues. This work includes peer-reviewed publications and models developed in the literature from October 2017 to January 2021. Information about the software, type of model, size, and population model was extracted for each article. In general, modeling was mainly done for DDI prediction via Simcyp^®^ software and Full PBPK. Overall, the necessary physiological and physio-pathological parameters, such as weight, BMI, liver or kidney function, relative to the drug absorption, distribution, metabolism, and elimination and to the population studied for model construction was publicly available. Of the 46 articles, 32 sensibly predicted DDI potentials, but only 23% integrated the genetic aspect to the developed models. Marked differences in concentration time profiles and maximum plasma concentration could be explained by the significant precision of the input parameters such as Tissue: plasma partition coefficients, protein abundance, or Ki values. In conclusion, the models show a good correlation between the predicted and observed plasma concentration values. These correlations are all the more pronounced as the model is rich in data representative of the population and the molecule in question. PBPK for DDI prediction is a promising approach in clinical, and harmonization of clearance prediction may be helped by a consensus on selecting the best data to use for PBPK model development.

## Introduction

In more cases than expected, the therapeutical management process involves a myriad of errors making drug-related problems (DRP) a recurring reviewed subject. In general, a large part of the DRP originates from drug prescribing issues (Perry et al., 2020). Difficulties can range from prescribing an inaccurate dose to inadequate administration frequency on top of a known allergy or a drug-drug interaction (DDIs). Among these risk factors, belonging to extremes of age, renal and liver impairment, or having genetic variations, are likely to increase developing DDI. Combination therapy is becoming increasingly prevalent in managing concurrent or single disease (Bi et al., 2018b), especially in geriatric patients. Previous Swiss studies have shown that polypharmacy prevalence was 11.8% and that it increased with age from 2.9% for age group 40–49 to 25.5% for age group 65–81 (Castioni et al., 2017). Dechanont et al. showed that DDI represents 1.1% of overall hospital admissions in this population and that 22.2% of ADRs are related to DDIs.

### Pharmacokinetic and Pharmacodynamic Drug-Drug Interactions

A pharmacokinetic (PK) DDI occurs when a perpetrator drug impacts the absorption, distribution, metabolism, or elimination (ADME) of a victim drug in one or more of the human body compartments. Pharmacodynamic (PD) interactions occur when two medicines directly interact (for example, on the same drug target) without altering the ADME parameters. PK and PD interactions may enhance activity (synergism) or decrease the effects (antagonism), affecting plasma drug levels and effects and having more or less severe consequences depending on the therapeutic margin of a drug (Hanke et al., 2018). The clinical consequences of DDIs can vary significantly in severity, from a simple rash to a life-threatening event or a serotoninergic syndrome (Prieto Garcia et al., 2018; Wang et al., 2019). The absorption of drugs and the ability to metabolize them varies considerably from one individual to another. The intrinsic difference between individual patients is caused by the inheritance of variant alleles, encoding drug-metabolizing enzymes. Genetic variations are estimated to contribute 20–30% of the variability in drug response (Sim et al., 2013).

### Drug Metabolism and Transport

**FIGURE 1:**
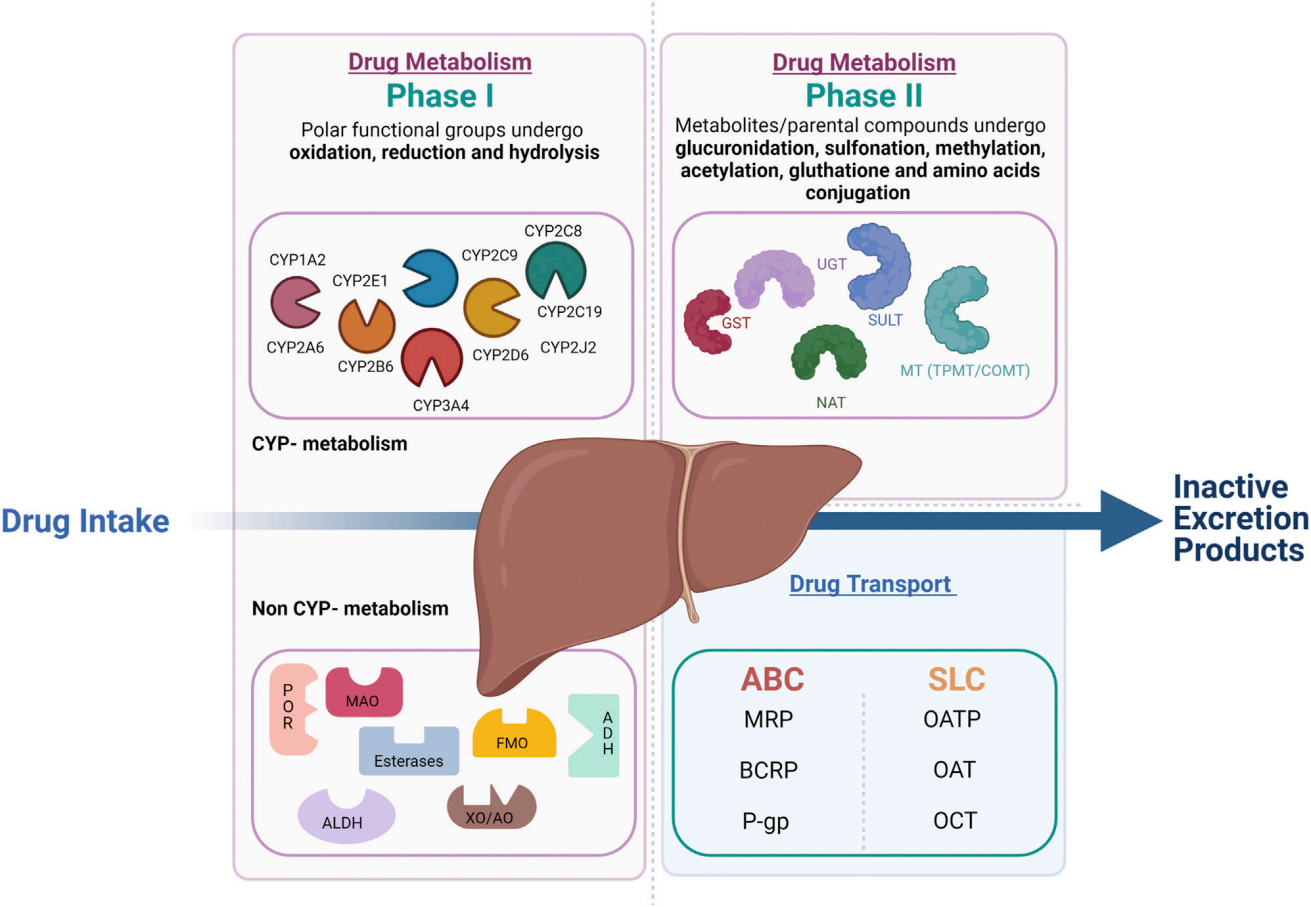
Overview of drug metabolism and transport in the liver. Drug metabolism is divided into phase 1 and phase 2 reactions. In phase 1 reactions, polar functional groups are unmasked or introduced to the molecules through oxidation, reduction and, hydrolysis. The so formed phase 1 metabolites can be readily excreted or can undergo subsequent conjugation reaction with hydrophilic moieties (phase 2 reactions). Transporters play a complemental role to the phase 1 and 2 assuring the phase 0 (uptake) and phase 3 (export) crucial to the drug elimination by metabolism.

Drug metabolism is divided into phase I and phase II reactions (Figure 1). Although most phase I metabolic reactions are catalyzed by Cytochrome P-450 (CYP450), the most studied metabolizing enzymes, other enzymes such as oxidoreductase, esterases, and oxidases can also be involved in phase I drug oxidation, reduction, and hydrolysis. Phase II reactions are conjugation reactions in which phase I metabolites or the parental compounds themselves undergo glucuronidation, sulfonation, methylation, acetylation, glutathione, and amino acids conjugation. The main enzymes involved in phase II drug metabolism include UDP-glucuronosyltransferases (UGTs), sulfotransferases (SULTs), N-acetyltransferases (NATs), glutathione S-transferases (GSTs), and various methyltransferases (MTs) (Oda et al., 2015) In parallel to the metabolic enzymes, membrane transporters also play a crucial role in drug absorption, distribution, and elimination. In complement to the metabolic phase I and phase II elimination, the term phase III elimination is sometimes used to describe the excretion of drugs and their metabolites by carrier-mediated uptake of drugs (Döring and Petzinger, 2014). Drug-transporters are membrane-bound proteins expressed in various organs and play an essential role in influencing drug absorption (phase 0) and elimination (phase III) of drugs and their metabolites and hence, therapeutic efficacy.

### Genetic Polymorphism of Drug Metabolizing Enzymes and Transporters

During the last decades, genes responsible for drug metabolism and transport and their most common functional variants have been identified based on the sampling of extreme phenotypes. For instance, for the CYP enzymes, four phenotypes with progressively increasing CYP activity can be defined: poor metabolizers (PMs) lacking the functional enzyme, normal metabolizers (NMs) homozygous for normal alleles, intermediate metabolizers (IMs) heterozygous for one deficient allele, or carrying two alleles that result in reduced activity and ultra-rapid metabolizers (UMs). The latter carry multiple gene copies (Ingelman-Sundberg 2005). Based on similarities of the sequence of genes encoding P450 enzymes, 18 CYP450 families are distinguished and branch out into 43 subfamilies (Waring 2020). All genes encoding P450 enzymes in families 1–3 are polymorphic (Zanger and Schwab 2013). Up to now, more than 350 functionally polymorphic CYPs (not counting the subvariants) that affect the function and/or activity of the gene products have been presented on the Human CYP allele nomenclature committee web page (http://www.imm.ki.se/cypalleles) (Zhou et al., 2009). The most important CYP families related to drug metabolism are CYP1A, CYP2C-D-E, and CYP3A. Interindividual variability considerably marks CYP1A2. Even though most of the CYP1A2 variability is due to genetic elements, this enzyme’s activity and expression are widely influenced by environmental factors. Cigarette smoking and excessive consumption of broccoli, among other things, are well-established CYP1A2 inducers (Anttila et al., 2003; Vanduchova et al., 2016). CYP2C8, CYP2C9, CYP2C18, and CYP2C19 are four highly homologous genes that distinguish CYP2C subfamilies. Of these four genes, CYP2C9 and CYP2C19, with a potential functional impact on the drugs’ efficacy and adverse effects, are the most clinically relevant. CYP2C9 is accountable for 15–20% of phase I metabolized drugs (Läpple et al., 2003). CYP2E1 is responsible for the metabolism of 2.5% of clinically relevant xenobiotics, mainly small molecules (Hines 2008). CYP2D6 is the most polymorphic metabolic enzyme, with over 145 different alleles to date (Gaedigk et al., 2017). CYP3A subfamily enzymes include CYP3A4, CYP3A5, CYP3A7, and CYP3A43. The first three shares 85% sequence similarity responsible for 46% of the oxidative metabolism of clinically relevant drugs (Williams et al., 2002). Besides the CYPs mediated–DDI, the DDIs may be related to non-CYP enzymes and transporters, the most important of which, UGTs, uptake transporters (OATPs, OATs, and OCTs), and efflux transporters (P-gp, BCRP). Comparably to CYPs, UGT is principally located in the liver but can also be found in other tissues. UGT1-UGT2 can be divided into 3 subfamilies UGT1A, UGT2A and UGT2B. The UGT1A1, 1A3, 1A4, 1A9, and 2B7 are the hepatic ones responsible for conjugating 80% of common drugs known to be glucuronidated. In addition, many drugs can act as UGT inhibitors or inducers (Uchaipichat et al., 2006; Aceves Baldó et al., 2013). Drug transporters are categorized into two superfamilies: solute carrier (SLC) and ATP-binding cassette (ABC). The SLC transporters are typically involved in the uptake of drugs into the cells across the basolateral membrane through facilitated diffusion or secondary active transport. ABC transporters are efflux transporters that utilize primary active transport. The well-known transporters involved in DDIs are P-gp, BCRP (ABC transporters), OATP1B1/OATP1B3, OAT1/OAT3, OCT2, and MATE1/2K (SLC transporters).

### Predictive Models

**FIGURE 2:**
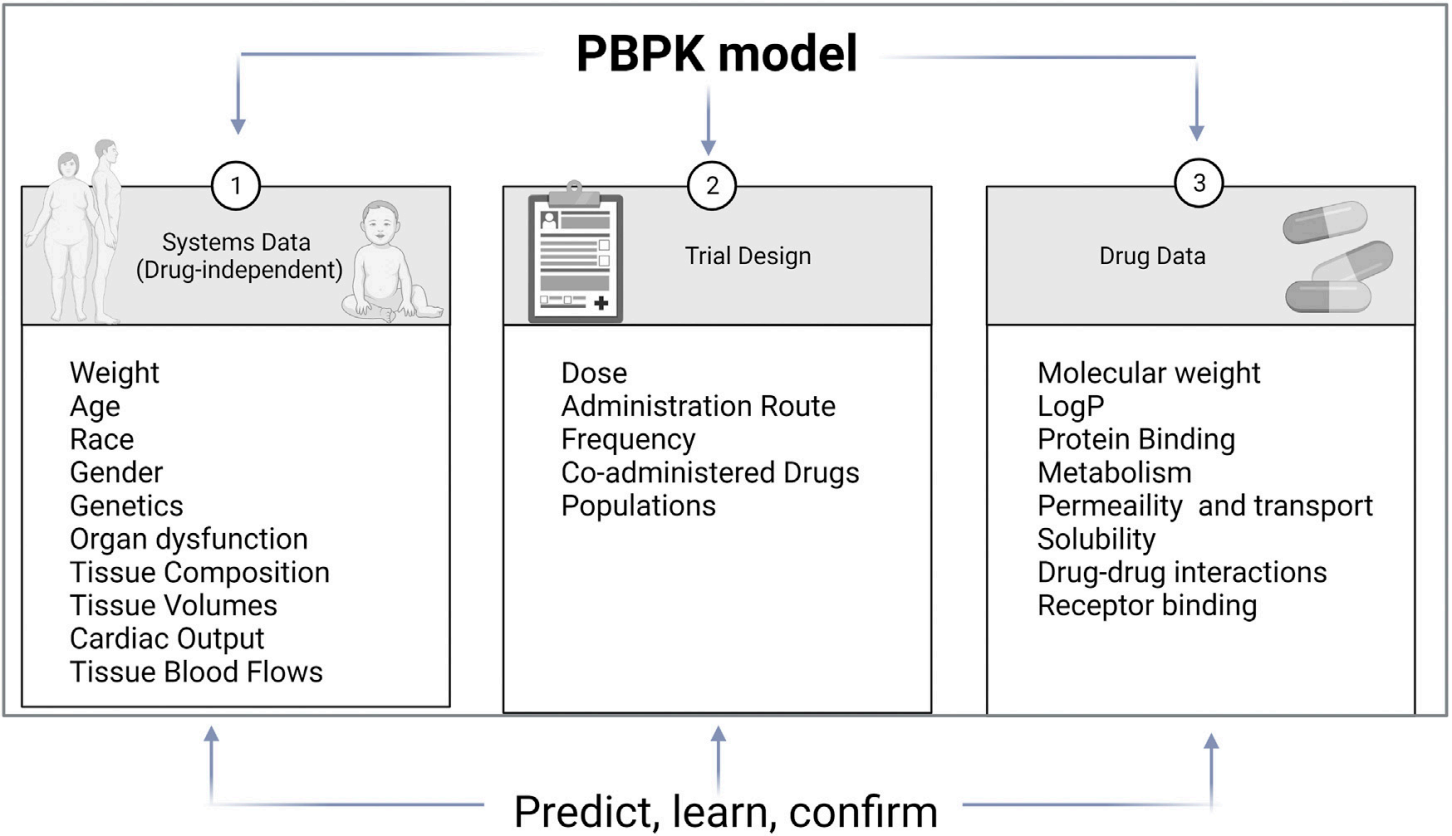
PBPK model components. PBPK models are separated into three main components: the drug, the system and the trial. Drug data include physicochemical and experimental or predicted ADME data. System data include physiological data which relevant for the ADME properties of drugs. Trial data include information on trial design such as administration route, dose regimen or trial duration (adapted from (Jamei 2016))

Improvement in computational tools led to predictive models used in clinical pharmacology. The main modeling approaches are quantification of structure-activity relationships (QSAR), quantitative systems pharmacology (QSP), and Pharmacokinetic modeling (PK modeling). QSAR is based on physicochemical and structural properties and identifies and explains intra- and inter-individual variability. QSP describes drug activity as a perturbation of a biological system. PK modeling aim to explain all PK characteristics of a drug and describe substrate’s and inhibitor’s, time-variable concentrations (Figure 2). Classical PK models are static mathematical models typically used to describe the relationship between the plasma or relevant tissue concentration of the drug and time. Over time, the classical approach based on a central compartment representing plasma linked to one or two peripheral compartments via rate constant evolved towards multicompartmental models referred to as physiologically based pharmacokinetic (PBPK) (Jones and Rowland-Yeo, 2013). Unlike other approaches, PBPK describe time-variable concentrations of the substrate in the different organs of the body. Comparatively to classical PK, it is a bottom-up, dynamic approach integrating drug-specific data and species physiology (system data, independent from the drug) to assess the impact of single and or combined intrinsic and extrinsic factors such as genetics, physiology, diseases, or co-treatments, on drug PK and PD properties in a population of individuals rather than an average subject. It divides the body into anatomically and physiologically meaningful compartments integrating system specificities and drug properties (Jones and Rowland-Yeo, 2013). PBPK models are built based on the same mathematical framework as classical PK models. PBPK numerous compartments correspond to the different organs or tissues in the body and incorporate biological and physiological components of each. These compartments include the central tissues of the body, namely, adipose, bone, brain, gut, heart, kidney, liver, lung, muscle, skin, and spleen, and are linked by the circulating blood system PBPK model structure is built upon the system properties composed of two parts, the anatomical one, and the drug-specific one. The system-related components consist of an anatomical part that describe the species-specific physiological parameters and a drug-specific part that describes the individual’s drug’s ADME properties (Jones and Rowland-Yeo, 2013). Therefore, the “system” operates the importance of demographic, anatomical, and physiological variables such as hepatic blood flow, CYP abundance, liver volume, and liver/renal function as a function of disease or age.

Building a “system” property to PBPK models allows for quantitative assessment of the impact of the covariates cited previously. PBPK modeling strategy relies on the iterative “Learn, confirm, and refine” approach (Darwich et al., 2017). The PBPK model is developed and validated in a healthy volunteer population starting from available data on the literature and/or on data collected in preclinical studies or from *in vitro* experiments. Following models building, simulations are run in the target population using relevant “system” and “drug” specific parameters. PBPK is an established tool that is now accepted by the regulatory authorities such as the Food and Drug Agency (FDA), European medicines agency (EMA), and Japanese Pharmaceuticals and Medical Devices Agency (PMDA) (Shekhani et al., 2020). It provides a mechanistic framework for predicting the time course of systemic and tissue exposure and drug response to various routes of administration and dosing regimens in different populations (age, gender, ethnic groups, healthy volunteers, diseases) (Manolis and Pons, 2009). Open platform initiatives such as PK-Sim and various PBPK platforms such as SimCYP^®^ and GastroPlus^®^ have been developed. They are user-friendly, do not require coding capabilities, and allow easy handling of physiology-based modeling (Rowland et al., 2011).

Despite significant scientific advances over the past 50 years and improved knowledge of enzymes and drug metabolism and disposition, DDI still represents an issue. In addition, many questions and challenges about the interplay between DDI and metabolic enzymes/transporter’s genetic variation arise. Therefore, it has become urgent for health to predict vulnerability to DDIs that cause adverse effects. Here we discuss the performance of the PBPK for predicting DDIs and the different sources of information used to build PBPK models to show what needs further investigation. Therefore, we have built a specific knowledge base to document predictions using PBPK, including peer-reviewed publications and models developed in the literature from October 2017 to January 2021. PubMed searches were conducted using “physiologically based pharmacokinetics” and “modeling” in the manuscript’s abstract or title. Articles were selected for review if published in English and focused on PBPK modeling applied to human pharmaceutical products. The publication was characterized according to the class of medicines to which it related. In addition, information about the software, type of model, size, and population model was extracted for each article (Supplementary Material). The final aim was to build a repository of available PBPK models developed to date to predict DDIs in the different therapeutic areas by integrating intrinsic and extrinsic factors such as genetic polymorphisms of the CYPs/transporters or environmental clues.

## Discussion

Related to what has been recently described in the literature (Min and Bae, 2017), a review of recent models suggests that the majority of PBPK models published after October 2017 are designed for the assessment of DDI (68%) followed by dose adaptation for pediatric, and then hepatic and renal failure. Most (73%) of the models were developed using the Simcyp simulator. One of the unique features of this software compared with other PBPK simulation software is that it predicts drug fate in an average population and in “outlier” individuals (Rostami-Hodjegan, 2012). Analysis of the published models also revealed that most of them were built according to a distribution model called full PBPK. This is an entirely mechanistic model where each organ is represented as a compartment instead of the simplified model. In the simplified model, the organs can be grouped into one or two symbolic compartments, called “minimal PBPK” (Kuepfer et al., 2016). An advantage of the full PBPK strategy is that it simulates the exposure of a drug or its metabolites in specific tissues that are not accessible to clinical sampling.

Additionally, depth analysis of the report pointed out the integration of the genetic aspect to the PBPK model in 23% of them. As described above, CYP450 isoenzymes are characterized by significant genetic polymorphism. Since CYP450 isoenzymes functionality is critical to its impact, genetic polymorphism may influence their magnitude (Tod et al., 2013). Although now incorporated in some guidelines, the impact of pharmacogenetic factors on the interaction between a drug and CYP450 isoenzymes (drug-gene interaction [DGI]) does not consider the change in the magnitude of the interaction depending on the genotype in question. This interaction is rarely considered in clinical practice, and systematic evidence of such critical pharmacogenetics effects on DDIs is lacking. Polymorphism also plays a crucial role in the metabolism of drugs with multiple metabolic pathways.

In this optic, Bi et al. (Bi et al., 2018b) assessed the role of previously unrecognized OAT2 transporter-mediated hepatic uptake in the pharmacokinetic of high permeability-low MW acidic and zwitterionic drugs (ECCS 1A) such as tolbutamide and warfarin. To do so, they selected 25 ECCS 1A drugs and tested transport activity using an *in-vitro* transport-transfected cell. The majority of drugs selected showed an active uptake by plated human hepatocytes. The data collected from *in-vitro* experiments were used to power supply the models with additional data related to the transport. The simulation was run considering the uptake transport alone, the metabolism alone, and the interplay between them. The transporter-enzyme interplay approach improved prediction accuracy compared to the other two approaches (average fold error = 1.9 and bias = 0.93). Bi and coauthors underscored the importance of transporters in evaluating and predicting the drug PK and suggest the lack of transporters consideration in the field.

A complementary analysis of the clinical PK-DDI studies by Huth et al. evaluated via a hybrid bottom-up and top-down strategy the effect of DDI inducers or inhibitors of the CYP3A and CYP2C9 enzymes on the systemic exposure of the immunosuppressant Siponimod (Huth et al., 2019). Clinical PK data from single and multiple ascending doses, absolute bioavailability, human ADME, and fluconazole DDI study results were used as bases in the PBPK model building. In addition, the model was verified by fitting the predicted and observed PK profiles. Simulated DDI potential of fluconazole (CYP3A4 and CYP2C9 inhibition effect) and rifampicine (CYP3A4 and CYP2C9 induction effect) on the systemic exposure of Siponimod after oral administration was compared with the respective clinical study. The Final PBPK model was used to assess Siponimod DDI potential as substrate at a steady state in the presence of specific CYP3A4/CYP2C9 inhibitors for six clinically relevant CYP2C9 genotypes. What has been highlighted by these simulations is that when CYP2C9 metabolic activity is decreased (as is the case for CYP2C9 PMs (*3/*3)), CYP3A4 becomes the primary pathway for drug clearance. Thus, the introduction of strong CYP3A4 inhibitors increases DDI risk in CYP2C9*3/*3 subjects compared to other genotypes. This illustrated the impact when both CYP2C9 and CYP3A4 pathways are less functional or inhibited. Moreover, Huth and co-authors with these findings laid the foundation for DDI drug labeling recommendations, as they established the relevant influence of CYP2C9 polymorphism on the DDI behavior of Siponimod.

Similarly, Gong et al. addressed, with a hybrid bottom-up and top-down full PBPK model, the case of BMS-823778, a potent and selective inhibitor of a microsomal enzyme regulating the tissue concentration of biologically active cortisol (Gong et al., 2018). *In vitro* permeability of BMS-823778 was determined in a Caco-2 cell bi-directional permeability assay and effective permeability was predicted with a Simcyp built-in algorithm based on *in vitro* Caco-2 permeability results. Tissue:plasma partition coefficients (Kp) in various organs including liver, kidney, spleen, adipose, bone, heart, gut, muscle and skin were directly taken from a rat tissue distribution study. Steady state volume of distribution (Vss) was predicted based on the individual input Kp values in the aforementioned tissues with a global Kp scalar of 1, using the Rodgers and Rowland method in Simcyp. All these physicochemical properties and ADME parameters were used to construct the initial model. BMS-823778s major metabolism pathway is CYP2C19, supported by other minor pathways, mainly CYP3A and UGT1A4 (Cheng et al., 2018). Comparative simulations leveraging available pharmacogenetics and PK from clinical studies in healthy subjects, Caucasian, Chinese and Japanese with various CYP2C19 and UGT1A4 functionality were performed to catch the inter-populational PK variability. The verified model was applied to simulate BMS-823778 PK and predict potential DDIs resulting from a CYP3A4 strong inhibitor in subjects with CYP2C19 and UGT1A4 genetic polymorphisms. The *in-vivo* clearance of BMS-823778 and CYP2C19 predicted phenotype were directly correlated. Described clinical pharmacogenetics studies did not demonstrate an impact of UGT1A4 polymorphism on BMS-823778PK. In contrast, the model described the PK profile in subjects with the predicted CYP2C19 PM phenotype and UGT1A4*1/*2 genotypes, who had a 50% increase in exposure BMS-823778 compared to those with normal UGT1A4 activity. With this particular example, the research group has once again demonstrated the place of pharmacogenetics in PBPK models development and the performance of predicting the magnitude of PK and DDI when it is challenging or not feasible in clinical settings.

This is particularly the case for pediatric populations, as illustrated by Zakaria et al. (Zakaria and Badhan, 2018). In African pediatric population groups, the study described an effective PBPK model for predicting the impact of dosage regimen alterations on target seven-day lumefantrine plasma concentrations involving the CYP2B6. A process of five stages was followed for model development, validation, and refinement. The authors started by applying the lumefantrine compound file to healthy, South African, and Ugandan populations and opposing the obtained results to PK data from clinical studies. The second step consisted of modeling lumefantrine-efavirenz interaction and comparison with clinical data of two published studies for validation and refinement. Following this, the model has been applied in the pediatric population and validated against clinical data. Finally, the polymorphic nature of CYP2B6 was also taken into account in the model. Therefore, this study focused on predicting the risk of efavirenz-mediated DDIs on lumefantrine pharmacokinetics in African pediatric population groups considering the polymorphic nature of CYP2B6. After predicting the risk, Zakaria and co-workers proposed adapting of the dosage regimen to avoid the observed phenomenon. Indeed, they demonstrated that an extension of the current artemether-lumefantrine treatment regimen from 3-days to 7-days would counteract the reduction in efavirenz metabolism common with the *6/*6 genotype and hence enhance the attainment of target day-7 lumefantrine concertation in both *1/*1 and *6/*6genotype groups, thereby reducing the risk of recrudescence.

As with CYP2B6, CYP2D6, the most polymorphic metabolic enzyme, is of significant interest when predicting the impact of genetic on the vulnerability and magnitude of DDI. Storelli et al. highlighted the pharmacogenetics testing significance by comparing the magnitude of predicted and observed CYP2D6 mediated DDIs in different CYP2D6 genotypes using PBPK modeling (Storelli et al., 2019). This work consisted of the first study evaluating the usefulness of PBPK in predicting gene-drug-drug interactions with specific CYP2D6 inhibitors and substrates. The group’s predictions and observations converged on the following rule of thumb: the higher the CYP2D6 activity, the greater the magnitude of the interaction. Authors faced underpredictions of the DDI when using the bottom-up approach on Simcyp with the experimental KI values in the case of duloxetine and paroxetine models. To solve this, they opted to optimize the models and used, for DDI modeling, KI values obtained from *in vivo* DDI studies, rather than *in-vitro* ones were analyzed and adapted via a sensitivity analysis. The new KI value was then verified with a set of independent DDI data (in-vivo optimized KI values). Through this work, the authors illustrate that *in-vitro* models, although beneficial for collecting information, may not describe specific mechanisms and therefore generate a margin of error in the prediction. Therefore, the comparison of simulated data with clinical data is crucial in the model’s validation and refinement. This study illustrates PBPK modeling performance in predicting of CYP2D6 genetic polymorphism effect on DDIs using verified initial models and rich PK from dedicated genetic trials to predict the effect of genotype on drug and substrate exposures.

Similarly, Chen et al. evaluated the systemic exposure of the tyrosine kinase inhibitor gefitinib in CYP2D6 UM and NM (Chen et al., 2018). Itraconazole DDI studies assessed the effect of the CYP2D6 genotype on gefitinib PK. Predictions showed that the gefitinib area under the curve (AUC) in CYP2D6 UM was reduced by 39% compared to NM. However, these changes were considered of limited impact because the reduced exposure was still above gefitinib *in vitro* IC_90_ for the patients of interest. Thus, the authors underline some challenges encountered with drugs identified as highly variable, like gefitinib, when it comes to PK and intersubject drug exposure. The present study demonstrated the unique potential of PBPK in predicting drug-drug interactions in pharmacogenomic subpopulations that could be hard to study due to low allele frequencies in a patient population. Authors suggest PBPK modeling as an alternative to conducting an actual clinical trial in these cases.

In a context of sinogliatin late-stage development and PBPK model development for study design and dose selection, Song et al. (Song et al., 2018) propose an effective strategy based on mechanistic insight into human drug metabolism and pharmacokinetic properties from preclinical *in vitro* and *in vivo* data using algometric scaling (AS), in vivo-to *in vitro* extrapolation (IVIVE) and steady-state concentration-mean residence time (Css-MRT). As described in the paper, The AS method provided the group for the model development with human clearance and steady-state volume distribution after intravenous administration. The IVIVE strategy allowed the verification of the *in-vitro* metabolic data and confirmed the predominant CYP enzyme involved in in vivo metabolism and corresponding fraction. Concerning the Css-MRT approach, it provided the knowledge on the interspecies difference that enabled selection of the optimal species to construct the preclinical PBPK model in some first in human studies. Instead of basing the model construction of literature research, authors implemented the available models with the collected parameters obtained from human major pharmacokinetic parameters analysis. The developed model successfully predicted human PK and evaluated the effects of extrinsic (e.g., DDI) and intrinsic (e.g., hepatic cirrhosis, CYP genetic) factors on drug exposure supporting the development of the drug candidate.

PBPK modeling is an assessed tool applicable to complex interactions investigation implying multiple drugs and genetic polymorphism, yet some authors propose its application for Physico-chemical DDI detection. The research article by Türk et al. (Türk et al., 2019) described, via whole-body PBPK models, CYP2C8 and organic-anion–transporting polypeptide (OATP) 1B1-based DDGIs involving the perpetrator drug gemfibrozil and the two victim drugs repaglinide and pioglitazone. PK-Sim and Mobi modeling software were used for the model development, and model construction relied on extensive literature research on the physicochemical and ADME processes of the drugs of interest. When available, system-dependent parameters were taken as provided by the simulation software; otherwise, they were collected from the literature. A total of 103 Clinical studies were digitalized from the literature and divided into an internal dataset for model building and parameter optimization and an external dataset for model evaluation. In the process of complex DDI modeling, the group demonstrated that a simultaneous administration of gemfibrozil might decrease the poor solubility of itraconazole, causing a decrease in absorption and thereby a decrease of the plasma concentrations of itraconazole and its metabolite. The same phenomenon was observed with pioglitazone when co-administered with gemfibrozil plus itraconazole. Through these two examples, the group illustrates that PBPK modeling is a valuable tool to develop and test hypotheses for unexpected clinical findings and raise awareness of the possibility of solubility interactions often put aside.

As a narrow therapeutic index drug, warfarin prescription demands a personalized medicine approach to tackle the interindividual variability and balance the therapeutic benefits and bleeding risk. Individualization is made based on genetic variants in CYP2C9 and vitamin K epoxide reductase (VKORC1). As stated by Bi et al. (Bi et al., 2018a), another specificity to this drug is that it is a racemic mixture of R- and S-enantiomers where CYP2C19 and other CYP enzymes metabolize R-warfarin, and S- warfarin is metabolized at 20% by CYP2C9. In this context and following this clinical observation, authors developed a bottom-up full PBPK model to evaluate the potential role of transporter-mediated hepatic uptake in the disposition of both warfarin enantiomers. The authors performed an *in-vitro* -in vivo extrapolation implementing the models with *in-vitro* obtained transporter kinetic data in primary human hepatocytes. Comparatively, to when OAT2-CYPs interplay was considered, when only CYP-mediated metabolism was assumed, authors faced an underprediction of oral clearance of both enantiomers. Despite the lack of clinical data needed to validate the model, the model developed with the OAT2-CYPs interplay recovered clinical pharmacokinetics, drug-drug- interactions, and CYP2C9 pharmacogenetics. Overall, Bi et al. have succeeded in demonstrating the utility of *in-vitro* data-informed- mechanistic modeling and simulations to enable the deconvolution of transporter-enzyme interplay and its role in governing drug pharmacokinetics, especially for untestable scenarios.

### Clinical Perspectives

As described above, a considerable part of xenobiotic biotransformation depends on the metabolizing enzymes and transporters. This has an impact not only on drug design but also on drug response. In this context, the regulatory authorities such as the FDA and the EMA have required systematic risk-based methodologies to evaluate drug parameters during the drug development process (Jamei, 2016). PBPK is used for mechanistic studies, aiding clinical development decisions, or drug discovery in the pharmaceutical industry. At the research and drug development, PBPK has already proven itself and is now an integral tool in drug discovery and development. It is a good tool for optimizing clinical trial designs, dose selection, and PK extrapolation from the general population to more specific ones. PBPK modeling can also be applied as an alternative to DDI trials in some special populations where actual DDI trials are hard to conduct due to logistical and ethical reasons (Huang et al., 2013). New drug application approval packages include preclinical and clinical investigation data. The potential effect of a new molecular entity on the metabolism or transport of other drugs, as well as the risk of being affected by other drugs, including recommended clinical index substrates and specific inhibitors or inducers of drug-metabolizing enzymes, are tested prior to the marketing authorization application. In addition to being used as the basis for new drug labels and summaries, the findings of those investigations are made available in the scientific handbooks and databases (Reis-Pardal et al., 2017). This provides healthcare prescribers and providers with the know-how to use the medicine safely and effectively. These data are also a primary source of information for PBPK simulation for treatment adaptation and dose prediction (Kuepfer et al., 2016). PBPK model’s part on the drug parameters is built and optimized to obtain the right absorption, distribution, metabolism, and elimination profiles. When physicochemical or ADME parameters are not available, they can either be predicted by the software according to implemented mathematical formulas or extrapolated from *in-vitro* model measurements (Emoto, Murayama et al., 2009). Different *in-vitro* systems are available to collect data and allow model enrichment to obtain the best predictive results. These systems include microsomes, recombinant enzymes, hepatocytes, and liver cells. Although different from each other, they all have the advantage of reducing the risk associated with potential adverse effects in humans, limiting costs, and having the potential for widespread use (Stillhart et al., 2019). The study population is critical in the prediction process, along with the parameters related to the molecule studied and the galenic formulation of interest. Therefore, different virtual populations have been developed and are available within the PK modeling software. A virtual population is characterized by its demographic parameters such as mean age, the proportion of females and males, but also by physiological and pathophysiological parameters (Hartmanshenn et al., 2016). Organ size, blood flow, and protein abundance parameters, for example, are data that are modified to represent the target population and best predict pharmacokinetics. More recently and with the emergence of knowledge in pharmacogenetics, many simulations have been performed during drug development to predict the vulnerability to DDI in groups of patients with different genotypes (Pastino et al., 2000; Wu et al., 2014; Djebli et al., 2015; Toshimoto et al., 2017). Faced with this advance, many groups are trying to apply the same principle to personalized medicine and are thinking of implementing a pharmacokinetic prediction model based on patient X-specific data within prescription support software. This means individualizing the drugs PK prediction PK by creating a computer model replicating the patients attributes able to affect drug exposure: virtual twin appraoch (Polasek et al., 2018).

Based on the drug and population parameters, PBPK aims to optimize individual drug dosing regimens and ensure therapeutics safety and efficacy. Other methods with the same goal are currently used in the clinic, including therapeutic drug monitoring (TDM). TDM is based on laboratory measurements of a chemical parameter in the patient’s biological fluids at crucial times to maintain drug concentrations within a targeted therapeutic window (Ghiculescu, 2008). This clinically implemented drug individualization approach, in contrast to PBPK, is a short-term solution to facilitate dosing and account for DDI. Although it compensates for inter and intra-individual variability in drug response, the measures implemented are only temporary and must be reevaluated for slight changes in intrinsic or extrinsic factors (Ghiculescu, 2008). However, when TDM is available, the generated data can be introduced into a PBPK model to make the prediction more robust. Thus, a multidisciplinary approach combining knowledge of pharmaceutics, pharmacokinetics, and pharmacodynamics is essential to predict the most appropriate drug response in specific individuals.

In a study by Glassman et al., clinical pharmacist’s detection of DDI on drug pairs was 44% (Glassman and Balthasar, 2019) and went up to 66% in another study by Weidemann et al. (Weideman et al., 1999). Despite the pharmacological knowledge of pharmacists and physicians, detection tools seem necessary to reduce DDIs, especially for new drugs on the market or complex treatments. Clinical decision support systems are the product of computerized physician order entry (CPOE) implementation combined with the transition from manual order entry to electronic health records (EHR). They have considerably improved the systematic screening and detection of DDIs and decreased prescribing problems and DDIs (Nuckols et al., 2014). Computerized systems implemented with decision-support provide automatic alerts to the prescriber based on analysis of clinical data in CPOE (Riedmann et al., 2011). Alerts can be related to clinical issues such as duplicate therapy, drug allergies, or potential DDIs. Although very advantageous, they have several limitations. First, it provides support only at the step of prescribing, taking into account relevant biochemical parameters in a minimal number of cases. In addition, it has been reported that this type of system generates “alert fatigues," causing them not to consider the recommendations issued by the program at all (Kuperman et al., 2007). For drugs for which polymorphic enzymes/transporters are the main clearance factors, PBPK simulations can be used to give a genotype-specific dose and dose adjustment recommendation. This would be an essential step in precision medicine without performing DDI studies for all the genotypes involved. Accordingly, integrating prediction software with prescribing support software may be of great benefit and a big step forward in personalized medicine (Venkatakrishnan and Rostami-Hodjegan, 2019).

## Conclusion

Current treatment regimens rely on the anticipated relationship between drug doses, plasma levels, and desired effect. Current perspectives in individualized therapy and personalized medicine aim to quantify anticipated changes in patients, evolving from prediction in general populations to individual patient responses and modeling. This review provides an overview of PBPK model development and its integration into the application for PK predictions and decision-making tools. Forty-six PBPK modeling papers on the prediction of DDI potentials were identified, and the advantages of PBPK modeling, including accounting for time-varying changes and inter-individual variability, were highlighted. In investigating DDI potentials using PBPK modeling, a limited number of drug-metabolizing enzyme-mediated DDIs has been considered by the published studies. Moreover, the simulations were performed mainly on healthy adult populations. Therefore, to broaden the scope of PBPK modeling in predicting DDIs, more information about the physiological properties of the organism and the incorporation of environmental and pathophysiological conditions into disease states is needed (Lenoir et al., 2020; Magliocco et al., 2020). In addition, it must be taken into account that the patient genetic makeup, concerning their drug-metabolizing enzymes and transporters, determines the relationship between drug doses and plasma concentration and thus therapeutic effect. However, many data are required to implement predictive systems in clinics, and genetic knowledge of CYP450 alone is insufficient to predict DDI. Despite its remaining challenges, PBPK for DDI prediction represents an excellent asset for regulatory authorities and drug development and a promising approach in clinical practice in the frame of model-informed precision dosing and individualized therapy.

## Abbreviations

DRP, Drug-related problems; DDI, Drug-drug interaction; PK, Pharmacokinetic; ADME, Absorption Distribution Metabolism or Elimination; PD, Pharmacodynamic; CYP450, Cytochrome P-450; UGTs, UDP-glucuronosyltransferases; SULTs, Sulfotransferases; NATs, N-acetyltransferases; GSTs, Glutathione S-transferases; MTs, Methyltransferases; PM, Poor metabolizers; NM, Normal metabolizers; IM, Intermediate metabolizers; UM, Ultra-rapid metabolizers; SLC, Solute carrier; ABC, ATP-binding cassette; QSAR, Quantification of structure-activity relationships; QSP, Quantitative systems pharmacology; PBPK, Physiologically based pharmacokinetic; FDA, Food and Drug Agency; EMA, European medicines agency; PMDA, Japanese Pharmaceuticals and Medical Devices Agency; DGI, Drug-gene interaction; TDM, Therapeutic drug monitoring; CPOE, Computerized physician order entry; HER, Electronic health records.
