# Supplementary material for: Reviewing Data Integrated for PBPK Model Development to Predict Metabolic Drug-Drug Interactions: Shifting Perspectives and Emerging Trends
Source: Front Pharmacol. 2021 Oct 28;12:708299. doi: 10.3389/fphar.2021.708299 (PMC8582169; doi:10.3389/fphar.2021.708299)
Supplement: Supplementary file 1 [file DataSheet1.PDF]

Compounnd studied	Clearance pathways investigated	Drugs for model performance evaluation	Aim	Drug for PK prediction	Full/ Minimal	Female Proportion	Age range	Population + size	Dosage regimen	Incorporation of genetics	Software	Citation
Atorvastatin<br>(atorvastatin lactone)	CYP3A4<br>OATP1BA	Itraconazole<br>Clarithromycine<br>Rifampicine<br>Cyclosporine	DDI study : Prediction of muscle tissue and plasma exposure changes	Fluconazole<br>Diltiazem<br>Cyclosporine<br>Palbociclib<br>Sacubitril<br>Gemfibrozil	Full	0/0.5/1	40-65	50 Sim healthy volunteer<br>(5 trials x 10 subjects)	Oral SD and MD	No	Simcyp	(Li, Yu et al. 2019)
Fimasartan	CYP3A4<br>OAT1/T3<br>OATP1B1/2B1<br>OCT1	-	DDI study : Investigation of combination therapy of fimasartan, amlodipine, and hydrochloro-thiazide potentials	Fimasartan + Amlodipine and Fimasartan + Hydrochlorothiazide	Minimal (Amlodopine)<br>Full (Fimasartan and Hydrochlorthiazide)	0	20-40	100 Sim healthy volunteer<br>(10 trials x 10 subjects)	Oral MD	No	Simcyp	(Rhee, Lee et al. 2018)
Bosentan	CYP3A4<br>CYP2B6	Tadalafil Warfarin	DDI study: Estimation of liver exposure based on observed systemic pharmacokinetics from clinical studies	Tadalafil<br>Warfarin	-	-	-	1000 Healthy volunteer	IV and Oral SD and MD	No	MATLAB	(Li, Niosi et al. 2018)
Rifampicine<br>Digoxin<br>Itraconazole<br>Clarithromycine<br>Midazolam<br>Alfentanil	CYP3A4<br>P-gp	Midazolam<br>Alfentanil<br>Digoxin<br>Itraconazole<br>Clarithromycin	Models Optimisation : Construction of important CYP3A4 and P-gp perpetrator and victim drugs that are all compatible, evaluated, and fit for use in PBPK DDI modeling.	Midazolam<br>Alfentanil<br>Digoxin<br>Itraconazole<br>Clarithromycin	Full	0	30-30	100 healthy male European individual	IV and Oral SD and MD	No	PK-Sim and MoBi	(Hanke, Frechen et al. 2018)
Tolbutamide	CYP2C9<br>OAT2	-	DDI study : Evaluate the role of transporter-enzyme interplay in tolbutamide pharmacokinetics	Sulfaphenozone<br>Fluconnazole<br>Cimetidine	Full	0.5	18-65	100 Healthy volunteer (10 trials x 10 subjects)	Oral	Yes<br>CYP2C9<br>*1/*1 - *1/*3 - *2/*3 - *3/*3	Simcyp	(Bi, Mathialagan et al. 2018)
Oxycodone<br>Fentanyl<br>Diazepam<br>Buprenorphin	CYP3A4<br>CYP2D6	-	DDI study: Study ofthe mechanism underlying the DDIs between opioids and benzodiazepines from the perspective of their pharmacokinetic (PK) interactions	Alprazolam<br>Diazepam<br>Midazolam<br>Triazolam	Minimal (Alpra /mida /triazolam)<br>Full (opioid and diazepam)	-	18-50	100 Healthy volunteer (10 trials x 10 subjects)	Oral	No	Simcyp	(Ji, Liu et al. 2019)
CAM2038 *	CYP3A4	Ketoconazole<br>Rifampin	DDI study: Prediction of DDI magnitude with strong CYP3A4 inducer or inhibitors	-	Minimal	-	-	120 Healthy volunteer<br>(10 trials x 12 subjects)	SC	No	Simcyp	(Liu and Gobburu 2018)
Clarithromycin<br>Quinidine<br>Paroxetine<br>Ciprofloxacin<br>Fluconazole<br>Rifampicin<br>Itraconazole<br>Ketoconnazole	CYP3A<br>CYP2C9/19<br>CYP2D6	-	Models Optimisation: Establishment of the confidence degree in tested CYP modulators and to outline their potential deficiencies.	Midazolam<br>Simvastatin<br>Alprazolam<br>Triazolam<br>Zolpidem<br>Quinidine<br>Repaglinide<br>Caffeine<br>Theophylline<br>Dextromethorphan<br>Nifedipine<br>Metoprolol<br>Desipramine<br>Imipramine<br>Omeprazole<br>Phenytoin<br>Warfarin	Minimal for all but full for rifampicin	Variable	Variable	Healthy volunteer	Oral	No	Simcyp	(Marsousi, Desmeules et al. 2018)
Valproic acid	CYP2C9<br>UGT2B7<br>UGT1A3/4	-	Model Development :Develop a novel mechanistic PBPK model for VPA.	Lorazepam<br>Phenytoine<br>Carbamazepine<br>Phenobarbital	Minimal	Matched to the clinical studies	Matched to the clinical studies	Healthy volunteer and pediatric and cirrhosis population IR: 100 (10 trials x 10 subjects), ER :25 subjects (5 trials × 5 subjects)-20 subjects (2 trials ×10 subjects) patients with cirrhosis	Oral (IR and ER)	No	Simcyp	(Conner, Nikolian et al. 2018)
14C-erythromycin	CYP3A4<br>P-gp<br>MRP2	-	Metabolism study: Incorporating 14CO2 production rates of the erythromycin breath test (ERMBT) to differentiate the contribution of metabolic and transporter pathways to erythromycin disposition into the PBPK model	-	Full	-	-	12 Healthy Subjects	IV	No	The R package	(Franchetti and Nolin 2019)
[illegible]			Models Optimisation: provide a comprehensive mechanistic [illegible]			[illegible]	Matched to [illegible]	[illegible]				[illegible]

Itraconazole OH-ITZ Keto-ITZ	CYP3A4	-	[illegible] PBPK model for itraconazole in order to increase the confidence in its drug-drug interaction (DDI) predictions.	Midazolam	Full	Matched to the clinical studies	[illegible] the clinical studies	100 Healthy volunteer (10 trials x 10 subjects)	Oral	No	Simcyp	(Prieto Garcia, Janzen et al. 2018)
Letermovir	CYP3A<br>CYP2C8<br>OATP1B1	-	DDI study:Study of relevant letermovir PK and DDIs in order to support US prescribing information in absence of clinical DDI studies.	Repaglinide<br>Atorvastatine<br>Rosiglitazone<br>Midazolam	Full	0.5	20-50	100 Healthy volunteer (10 trials x 10 subjects)	Oral and IV SD and MD	No	Simcyp	(Wang, Chen et al. 2019)
Rilpivirine<br>Cabotegravir	CYP3A4	-	DDI study:DDI evaluation between LA (cabotegravir and rilpivirine) antiretroviral agents and rifampicin	Rifampicine	Full	0.5	18-60	Healthy volunteer	Oral and IM	No	Simbiology	(Rajoli, Curley et al. 2019)
Ritonavir	CYP3A4<br>CYP2C9<br>CYP2D6	-	DDI study : Evaluation of ritonavir effect at steady state on midazolam (CYP3A4 substrate) AUC	Midazolam	Minimal	Matched to the clinical studies	Matched to the clinical studies	Matched to the clinical studies	Oral	No	Simcyp	(Umehara, Huth et al. 2018)
Alectinib	CYP3A4<br>CYP2C8	Repaglinide	DDI study : Study of the potential DDI on repaglinide AUC	Posacoazole<br>Rifampicine<br>Midazolam	Minimal	-	-	60 Healthy and Cancer population (NSCLC: non-small cell lung cancer) (10 trials x 6 subjects)	Oral	No	GastroPlus and SimCYP	(Cleary, Gertz et al. 2018)
Baricitinib	OAT3<br>MATE2<br>P-gp	Ibuprofen<br>Diclofenac	DDI study: Determination the potential for clinical DDIs with concomitant therapies to baricitinib that inhibit renal secretion.	Probenecid	Full	0	22-63	North european white men	Oral	No	Simcyp	(Posada, Cannady et al. 2017)
Bosutinib	CYP3A4<br>P-gp	Itraconazole<br>Verapamil	Model optimisation- DDI study: setting of pgp on the model and prediction of bosutinib DDIs with ketocoazole and rifampicin	Ketoconazole<br>Rifampin	Full	0.5	20-50	36 Healthy volunteer (6 trials x 6 subjects)	Oral	No	Simcyp	(Yamazaki, Loi et al. 2018)
Dabrafenib	CYP2C8<br>CYP3A4	Ketoconazole Gemfibrozil Rifampin	Molecule Investigation: identification of biochemical and physiological characteristics that drive BSV in dabrafenib exposure.	-	Full	0.48	20-50 control. 29.2-87.8 cancer patients	Matched to the clinical studies	Oral	No	Simcyp	(Rowland, van Dyk et al. 2018)
Erlotinib	CYP3A4<br>CYP1A2<br>CYP1A1	-	Metabolism study : Asessing possible alterations in Erlotinib plasma concentrations in pancreatic cancer patients	-	Minimal		60 single 50-70 population	Healthy volunteer 25 (1 trial x 25 subjects)	Oral	No	GastroPlus	(Gruber, Czejka et al. 2018)
Getifinib	CYP2D6<br>CYP3A4	Itraconazole<br>Metoprolol	DDTGI study: Build a gefitinib PBPK model to simulate the pharmacokinetics in CYP2D6 UM and provide quantitative assessment of the systemic exposure in CYP2D6 UM vs EM	-	Minimal	Matched to the clinical studies	Matched to the clinical studies	Matched to the clinical studies	Oral	Yes CYP2D6 UM vs EM	Simcyp	(Chen, Zhou et al. 2018)
Ixazomib	CYP3A	Clarithromycin	DDI study : Evaluation of Ketoconazole, Clarithromycin, and Rifampin on Ixazomib PK	Ketoconazole<br>Clarithromycin<br>Rifampicin	Full	0.44	23-86	For each DDI study (10 trials x 16 subjects)	Oral	No	Simcyp	(Gupta, Hanley et al. 2018)
Midostaurin<br>CGP52421<br>CGP62221	CYP3A4/5	Ketoconazole<br>Midazolam<br>Rifampicin	DDI study: Predict potential DDI effects of CYP3A4 inhibitors or inducers on midostaurin and its metabolites as well as effects on midazolam under steady-state conditions.	Fluconnazole<br>Itracoanzole Ketoconazole<br>Efavirenz<br>Rifampicin Midazolam	Minimal	0.5	20–55	Healthy volunteer and Patients with (AML) and (advSM) 100 (10 trial x 10 subjects)	Oral Single and Multiple	No	Simcyp	(Gu, Dutreix et al. 2018)
Olaparib	CYP3A4<br>P-gp<br>UGT1A1	Itraconazole or Rifampicin	DDI study: Evaluation of Ddi effect on Olaprib PK	Itraconazole<br>Fluconazole<br>Fluvoxamine<br>Rifampicine<br>Efavirenz<br>Dexamethasone<br>Midazolam<br>Simvstatine<br>Digoxine<br>Raltegravir	Full	-	Matched to the clinical studies	100 Oncology population And healthy volunteers (10 trial x 10 subjects) and 140 for itraconazole (5 trials x 28 patients)	Oral Single and Multiple	No	Simcyp	(Pilla Reddy, Bui et al. 2019)
Osimertinib	CYP3A<br>BCRP	Itraconazole<br>Rifampicin<br>Simvastatin<br>Rosuvastatin	DDI study : Simulation of Osimertinib PK changes in the presence of weak to moderate CYP3A inducers commonly used in the clinics dexamethasone and efavirenz	Dexamethasone<br>Efavirenz	Full	-	-	100 Sim Healthy subjects (10 subjects of 10 trials)	Oral MD	No	Simcyp	(Pilla Reddy, Walker et al. 2018)
Panobinostat	CYP3A4<br>CYP2C19<br>CYP2D6	Ketoconazole	DDI study : Prediction of the interaction with the strong CYP3A4 inducer rifampin (RIF) and the sensitive CYP3A4 substrate midazolam (MDZ) in lieu of clinical trials	Rifampicine<br>Midazolam	Minimal	0.5	-	Simcyp "healthy volunteer" population Ten trials of 10 subjects	Oral SD and MD	No	Simcyp	(Einolf, Lin et al. 2017)
Ribociclib	-	-	Molecule investigation : Evaluation of the effect of changes in gastric pH on ribociclib absorption	-	Minimal	Matched tot the clinical study	Mathed to the clinical study	Healthy volunteer Matched to the clinical study	Oral SD	No	GastroPlus and SimCYP	(Samant, Dhuria et al. 2018)
Ruxolitinib	CYP3A4<br>CYP2C9	Fluconazole<br>S-warfarin<br>Tolbutamide<br>Phenytoin	DDI study: Estimation of Ruxolitinib victim DDI risks in the presence of CYP inhibitors	Ketoconnazole<br>Erythromycin<br>Rifampicine	Full	Matched tot the clinical study	Mathed to the clinical study	100 Sim Healthy subjects (10 subjects of 10 trials)	Oral SD and MD	No	Simcyp	(Umehara, Huth et al. 2019)
			Molecule investigation : Development of model to describe [illegible]				Mathed to [illegible]					(Emami [illegible]

Venetoclax	CYP3A4/5	Ketoconazole Rifampicine	Molecule investigation : Development of model to describe absorption and disposition of an amorphous solid dispersion formulation ofvenetoclax in humans	-	Full	1	Mathed to the clinical study	Sim Healthy volunteer (10 trials of 12 healthy volunteers)	Oral	No	Simcyp	Riedmaier, Lindley et al. 2018)
Zoptarelin doxorubicin	OAT1B3 OCT2 P-gp BCRP OAT1 OAT3 OATP1B1	-	DDI study: Establish a model of zoptarelin doxorubicin and apply it for drug–drug interaction (DDI) potential analysis.	Simvastatin Metformine	Full	Matched tot the clinical study	Mathed to the clinical study	Cancer patient population	Intravenous	No	PK-Sim and MoBi ad MATLAB	(Hanke, Teifel et al. 2018)
AZD1775	-	-	Molecule Investigation : prediction of plasma and brain concentration-time profiles of total and unbound AZD1775	-	full	-	-	virtual Caucasian population 10 trials with 10 subjects	Oral	No	Simcyp	35
Ethinylestradiol	CYP3A4 CYP1A2 UGT1A1	Ketoconazole	DDI study : Prediction of the effect of CYP3A4 inhibition and induction on the pharmacokinetics of EE	fluconazole voriconazole caramazepine rifampicine	Minimal	Mathed to the clinical study	Mathed to the clinical study	Mathed to the clinical study	Oral	No	Simcyp	(Ezuruike, Humphries et al. 2018)
Mirabegron	CYP3A4 UGT2B7	Construction : Ketoconazole Verification : Rifampicin and desipramine (As substrate)	DDI study : Evaluation of the magnitude of the DDI with ketoconazole taking into account the overall elimination pathways	Probenecid fluconazole	Minimal	0.5	20-50	200 Sim-Healthy volunteer	Oral	No	Simcyp	(Konishi, Minematsu et al. 2019)
Atomexetine	CYP2D6 CYP2C19 CYP3A	-	Molecule investigation : Prediction of Atomoxetine disposition in specific populations and in organ impairment patients	fluoxetine paroxetine desipramine midazolam	full	Matched the reported clinical study data	Matched the reported clinical study data	Healthy volunteer, japanese, chinese, pediatric and organ impaired populations	IV and Oral	Yes CYP2D6 EM vs PM	Simcyp	34https://doi.org/10.1124/dmd.117.076455
Dextromethorphan Tolterodine Risperidone	CYP2D6	-	DDTGI study: Prediction of the extent of Cytochrome P450 2D6 (CYP2D6)-mediated drug–drug interactions (DDIs) in different CYP2D6 genotypes using PBPK	Duloxetine Fluoxetine Paroxetine	Full	Matched the reported clinical study	Matched the reported clinical study	Healthy Volunteer population	Oral	Yes 1AS3 9AS2 14AS1 1 AS0.5 CYP2D6	Simcyp	(Storelli, Desmeules et al. 2019)
Gemfirozil Repaglinide Pioglitazone	CYP2C8 OATP1B1	-	DDTGI study: Evaluation of important cytochrome P450 (CYP) 2C8 perpetrator and victim drugs for DDI and DGI studies.	Rifampicine Clarithroimycine Itraconnazole	Full	Mathed to the clinical study	Mathed to the clinical study	Mathed to the clinical study	Oral	Yes CYP2C8 *1/*1, *3/*3 ; SLCO1B1 wild type and 521CC	PK-Sim® and MoBi®	(Türk, Hanke et al. 2019)
Sinogliatin	CYP3A4	-	DDTGI study:: supporting a clinical drug–drug interaction (DDI)study design to evaluate the effects of intrinsic (hepaticcirrhosis, genetic) factors on drug exposure.	itraconazole:	Full	Mathed to the clinical study	Mathed to the clinical study	'Chinese healthy volunteers' (n = 594)	Oral	Yes CYP3A4*1, CYP3A4*16 , and CYP3A4*18	Simcyp	(Song, Zhang et al. 2018)
Ethionamide	FMO3	-	DDTGI study : Assessing the effect of DDI-mediated metabolism on ethion-amide disposition	Metamizole	Minimal	0.5	18-50	Virtual healthy population (10 trials of 10 subjects)	Oral SD and MD	Yes FMO3 wild-type (Glu 158 or "H") or mutant (Lys 158 or "h") allele in exon 4 and the wild-type (Glu308 or "D") or mutant (Gly308or "d") allele in exon 7	Simcyp	(Nguyen, Parvez et al. 2019)
Midazolam S-warfarin metoprolol omeprazole lorazepam rosuvastatin	CYP3A4/5 BCRP CYP2C9 CYP2C19 CYP2D6 UGT2B7 UGT2B15 OATP1B1	-	Population study: development of a Korean-specific virtual population for the SimCYP® Simulator and evaluation the population's predictive performance using six substrate drugs	-	Full	0.5	20–50	400 virtual Caucasians, Japanese, Chinese (general)	IV and PO	Yes ?	Simcyp	(Kim, Hatley et al. 2019)

Siponimod	CYP2C9<br>CYP3A4	Fluconazole<br>Efavirenz<br>S-warfarin<br>tolbutamide<br>phenytoin	DDTGI study: Siponimod PK Prediction in presence of cytochrome P450 (CYP)2C9/CYP3A4 inhibitors/inducers in subjects with different CYP2C9 genotypes	Itraconazole<br>Ketoconazole<br>Erythromycin<br>Fluvoxamine<br>Rifampin	Full	Matched the reported clinical study	Matched the reported clinical study	Sim Healthy volunteers	Oral	Yes CYP2C9 genotypes (*1/*1, *1/*2, *1/*3, *2/*2, *2/*3, and *3/*3)	Simcyp	(Huth, Gardin et al. 2019)
Siponimod	CYP2C9	-	DDTGI study: Prediction of the inhibitory effects of fluconazole as well as the impact of cytochrome P450 (CYP) 2C9 genetic polymorphism on siponimod PK and metabolism	Fluconnazole	Full	0.5	18–65	Sim healthysubjects with the CYP2C9*1/*1 genotype	Oral SD and MD	Yes CYP2C9 genotypes (*1/*1, *1/*2, *1/*3, *2/*2, *2/*3, and *3/*3)	Simcyp	(Jin, Borell et al. 2018)
Midazolam<br>Tacrolimus	CYP3A4/5	-	DDTGI study: Prediction of CYP3A-mediated DDIs by integrating LC/MS measured	Ketoconazole	Minimal	0.5	20-50	Sim healthy volunteers	Oral	Yes CYP3A5 EM -PM	SimCYP	(Guo, Lucksiri et al. 2020)
Digoxin Metformin	MDR1 OCT1	-	Molecule investigation :Evaluation of the effects of decreased OCT1 levels on metformin	-	Full	Matched the reported clinical study	Matched the reported clinical study	Sim healthy volunteers	Oral for Metformin – IV for digoxin	Yes	SimCYP	(Ito, Sjöstedt et al. 2020)
Acetaminophe	UGT1A1 UGT1A6<br>UGT1A9 UGT2B15	-	Population study : Prediction of acetaminophen PK profile in the pediatric population.	-	Full	0	30	Healthy Caucasian	IV for constructionn and oral for validation	Yes	GastroPlus	(Ladumor, Bhatt et al. 2019)
BMS-823778	CYP3A4<br>CYP2C19<br>UGT1A4	-	DDTGI study: prediction of PK in subjects with multiple polymorphic enzymes and extent of DDI when coadministered with a strong inhibitor of CYP3A4	Itraconazole	Full	0	20-55	Sim healthy subjects (mainly Caucasian), Chinese and Japanese subjects with various CYP2C19 and UGT1A4<br>(10 trials with 10 subjects)	Oral Once daily	Yes CYP2C19 and UGT1A4 EM, IM and PM	SimCYP	(Gong, Iacono et al. 2018)
R- and S-Warfarin	CYP2C9<br>CYP2C19<br>CYP1A2	-	DDTGI study: capture of intestinal absorption and predict oral pharmacokinetics of R- and S-warfarin.	Amiodarone<br>Rifampicin	Full	-	18-65	Sim healthy subjects<br>(10 × 10 trials)	Oral SD	Yes CYP2C9 *1/*1,*1/*3, *2/*3, and *3/*3	SimCYP	(Bi, Lin et al. 2018)
Efavirenz	CYP2B6	-	DDTGI study: Prediction of the impact of efavirenz-mediated DDIs on lumefantrine pharmacokinetics inAfrican paediatric population groups	Lumefantrine	-	0.5	6 to 7	Healthy Volunteer (Caucasian), South African and Ugandan population	Oral MD	Yes CYP2B6 *1/*1,*6/*6	SimCYP	(Zakaria and Badhan 2018)

* (extended-release formulation of buprenorphine)
